# Prognostic value of lipoprotein (a) level in patients with coronary artery disease: a meta-analysis

**DOI:** 10.1186/s12944-019-1092-6

**Published:** 2019-07-08

**Authors:** Zhimiao Wang, Xincheng Zhai, Mei Xue, Wenjuan Cheng, Hesheng Hu

**Affiliations:** 1 Department of Cardiology, Shandong Provincial Qianfoshan Hospital, the First Hospital Affiliated with Shandong First Medical University, NO. 16766, Jingshi Road, Jinan city, Jinan, 250014 Shandong Province China; 2 Department of Geriatrics, Municipal Hospital of Zibo City, Zibo City, 255000 Shandong Province China

**Keywords:** Coronary artery disease, Prognosis, Mortality, Meta-analysis

## Abstract

**Background:**

Elevated lipoprotein (a) is recognized as a risk factor for incident cardiovascular events in the general population and established cardiovascular disease patients. However, there are conflicting findings on the prognostic utility of elevated lipoprotein (a) level in patients with coronary artery disease (CAD).Thus, we performed a meta-analysis to evaluate the prognostic value of elevated lipoprotein (a) level in CAD patients.

**Methods and results:**

A systematic literature search of PubMed and Embase databases was conducted until April 16, 2019. Observational studies reporting the prognostic value of elevated lipoprotein (a) level for cardiac events (cardiac death and acute coronary syndrome), cardiovascular events (death, stroke, acute coronary syndrome or coronary revascularisation), cardiovascular death, and all-cause mortality in CAD patients were included. Pooled multivariable adjusted risk ratio (RR) and 95% confidence interval (CI) for the highest vs. the lowest lipoprotein (a) level were utilized to calculate the prognostic value. Seventeen studies enrolling 283,328 patients were identified. Meta-analysis indicated that elevated lipoprotein (a) level was independently associated with an increased risk of cardiac events (RR 1.78; 95% CI 1.31–2.42) and cardiovascular events (RR 1.29; 95% CI 1.17–1.42) in CAD patients. However, elevated lipoprotein (a) level was not significantly associated with an increased risk of cardiovascular mortality (RR 1.43; 95% CI 0.94–2.18) and all-cause mortality (RR 1.35; 95% CI 0.93–1.95).

**Conclusions:**

Elevated lipoprotein (a) level is an independent predictor of cardiac and cardiovascular events in CAD patients. Measurement of lipoprotein (a) level has potential to improve the risk stratification among patients with CAD.

**Electronic supplementary material:**

The online version of this article (10.1186/s12944-019-1092-6) contains supplementary material, which is available to authorized users.

## Backgrounds

Coronary artery disease (CAD) is a common type of cardiovascular disease. Despite advances in medical sciences, CAD remains the main cause of death in the world [1]. Patients with CAD are facing high risk of secondary cardiovascular events and mortality. Nevertheless, current traditional risk factors and prognostic risk models are insufficient to predict the prognosis of CAD. Thus, identification of residual predictive biomarkers is crucial for more aggressive secondary prevention of CAD patients [2].

Lipoprotein (a) is a large low-density lipoprotein like particle involved in lipid metabolism, coagulation and fibrinolytic systems [3]. Approximately 20 to 30% of the global population have lipoprotein (a) level over 30 mg/ml a), particularly in patients with established cardiovascular disease [4]. Elevated lipoprotein (a) level or hyperlipoproteinemia (a) has been identified as a risk factor for incident cardiovascular events in the general population and established cardiovascular disease patients [5]. Blood lipoprotein (a) level was strongly associated with the presence and severity of CAD [6]. Accumulating epidemiological studies [7–18] suggest that elevated blood lipoprotein (a) level could serve as an independent prognostic biomarker in patients with CAD. However, there are conflicting results [19–23] on the prognostic role of elevated blood lipoprotein (a) level in CAD patients.

No previous systematic review or meta-analysis has focused on the prognostic value of elevated lipoprotein (a) level for mortality in patients with CAD. Therefore, this meta-analysis sought to investigate the prognostic value of baseline blood lipoprotein (a) level for cardiovascular and all-cause mortality and adverse cardiovascular events in CAD patients.

## Methods

### Literature search

This meta-analysis follows the guideline of Preferred Reporting Items for Systematic Reviews and Meta-analyses [24]. A systematic literature search of Pubmed and Embase databases was conducted by two independent authors until April 16, 2019. The search keywords included: “lipoprotein(a)” AND “coronary heart disease” OR “coronary artery disease” OR “myocardial infarction” OR “acute coronary syndromes” OR “unstable angina” AND “mortality” OR “death” OR “events” OR “event” AND “follow-up”. In addition, reference lists of included studies and pertinent articles were also manually scanned for any possible studies.

### Study selection

Studies were considered for inclusion if they satisfied the following criteria: 1) observational studies enrolling CAD patients; 2) baseline blood lipoprotein (a) level as exposure; 3) reported at least one of the following outcome measure: cardiac events (cardiac death and acute coronary syndrome[ACS]), cardiovascular events (death, stroke, ACS or coronary revascularisation), cardiovascular death, and all-cause mortality; and 4) provided multivariable adjusted risk ratio (RR) or hazard ratio (HR) or odds ratio (OR) with corresponding 95% confidence interval (CI) of each outcome measure for the highest versus the lowest blood lipoprotein(a). Exclusion criteria were: 1) participants were not restricted in CAD patients; 2) provided risk estimate according to per unit or per SD increase lipoprotein(a) level; 3) reported unadjusted risk estimate; and 4) meeting abstracts or reviews.

### Data extraction and quality assessment

Two authors independently extracted and collected the following data: last name of the first author, publication year, country, study design, type of CAD, sample size, percentage of men, mean age or age range, cutoff value of lipoprotein(a), length of follow-up, number of adverse events, fully adjusted risk estimate reported, and adjustment of variables. We assessed the methodological quality of individual studies based on the Newcastle-Ottawa Scale (NOS) for cohort studies [25]. Studies with 7 points or more were recognized as high quality. Disagreements between the authors in data extraction and quality evaluation were resolved by consensus.

### Statistical analysis

All data were analyzed using STATA 12.0 version software (STATA Corp LP, College Station, TX, USA).The reported multivariable adjusted risk estimate was used to calculate the prognostic value of blood lipoprotein(a) for the highest versus the lowest level. Heterogeneity between studies was checked using the I^2^ statistic (I^2^ ≥ 50% indicating significant heterogeneity) and the Cochrane Q statistic (*p* < 0.10 indicating significant heterogeneity). A fixed-effect model was chosen in the absence of significant heterogeneity. Otherwise, a random effect model was used. Funnel plot was scheduled to detect publication bias when more than 10 studies were analyzed. Moreover, we conducted a subgroup analysis by study design, sample sizes, follow-up duration, and whether adjusting statins use or lipids. Sensitivity analysis was performed by removal of one study at each turn to investigate the robustness of the pooling results.

## Results

### Search results and study characteristics

**Fig. 1:**
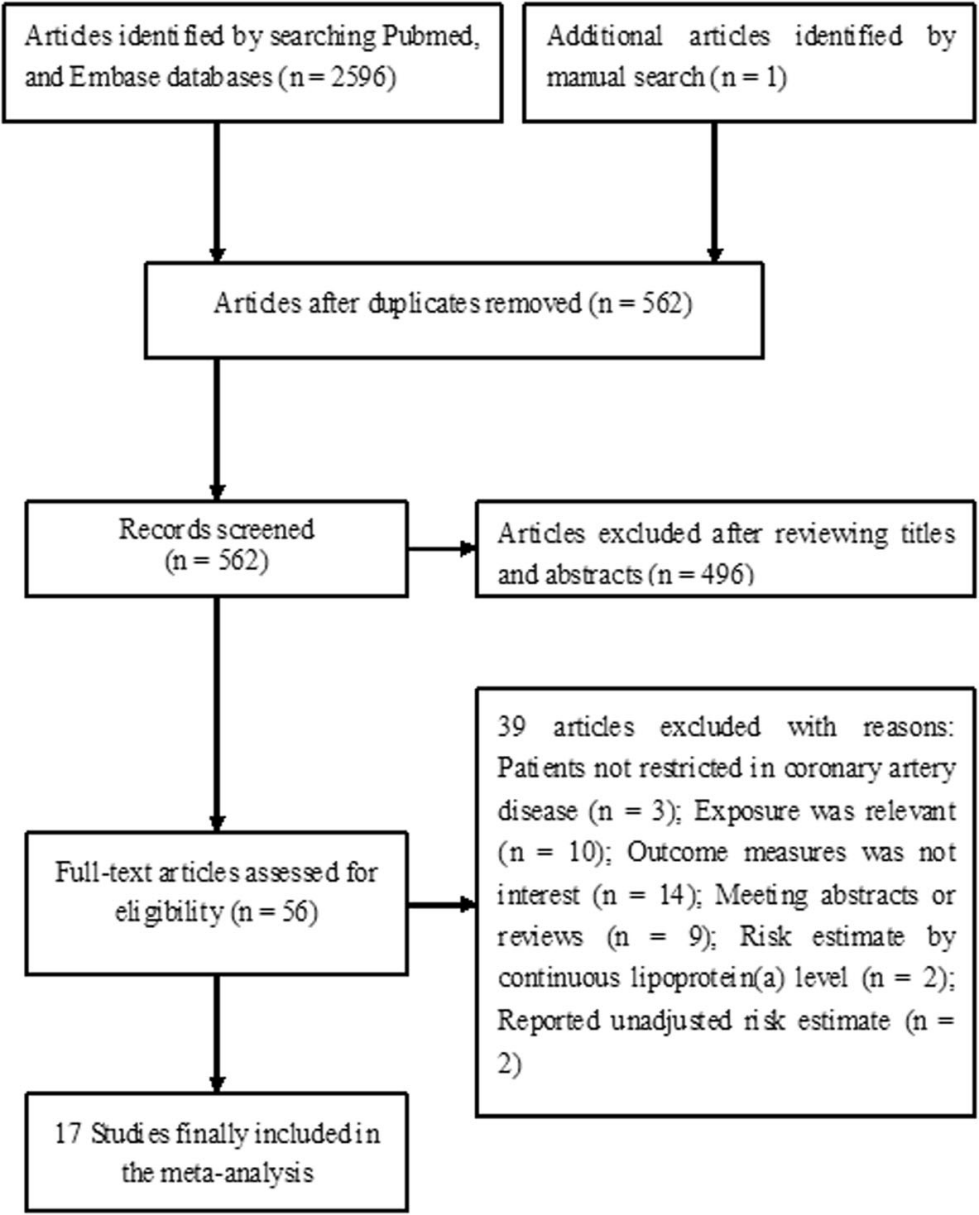
Flow chart showing study selection process

The study selection process is shown in Fig. 1. Our initial computerized literature search produced 2596 relevant records and one article was identified by a hand search. After reviewing titles and abstracts, 56 potentially relevant articles were retrieved for full-text evaluation. After applying our inclusion criteria, 39 articles were excluded for the following reasons: exposure were not lipoprotein (a); outcome measures was not interest; patients not restricted in CAD; meeting abstracts or reviews. Finally, 17 studies [7–23] were included in this meta-analysis.

Table 1 summarizes the main characteristics of these eligible studies. Ten studies [7, 8, 10, 12, 14–16, 19–21] adopted the retrospective designs and others were prospective. The included studies were published between 1998 and 2019. The sample size ranged from 115 to 7863, with a total of 283,328 patients. The length of follow-up ranged from 1.0 to 9.9 years. The cutoff value of lipoprotein varied across the included studies. Mortality events were evaluated by medical record or death certificate. Overall, there were 483 cardiac events, 3733 cardiovascular events, 1269 all-cause and 868 cardiovascular death in the included studies. Fifteen studies were awarded at least 7 points (Additional file 1 Table S1), indicating a relatively high quality.

**Table 1:** Basic characteristics of the included studies

Author/year	Country	Study design	Patients (% male)	Mean age (years)	Outcome definition	Lipoprotein (a) cutoff (mg/dL)	Outcome measures HR or RR(95% CI)	Adjustment for variables	Follow-up (years)
Stubbs 1998 [7]	UK	P	ACS;463 (73.8)	62.1 ± 11.1	–	≥30 vs. < 30	CV death:88 2.30 (1.34–3.94)^	Age, previous MI, infarct size, and hypertension	3.0
Shlipak 2000 [8]	USA	P	CAD;1383(0)	66.7	Cardiac death and non-fatal MI	> 55 vs. < 7.0	CE:182; 1.54 (1.0–2.4)	Race/ethnicity, DM, waist-to-hip-ratio, tobacco use, HDL-C, TG, and use of lipid-lowering agents, aspirin,and CCB.	4.1
Glader 2002 [9]	Sweden	P	CAD; 1216 (82)	59.4 ± 8.1	–	> 30 vs. ≤30	Total death:200; 1.4 (1.1–1.9); CV death:152 1.4 (1.0–2.0)	Age, gender, BMI, MCOS, LVMS, previous MI, hypertension, DM, history of stroke or claudication, present smoking, TC, HDL-C, TG, fibrinogen, antithrombin III, and ESR	6.7
Cho 2010 [19]	Korea	R	Post-acute MI; 832 (72.1)	62.8 ± 12.4	Total death, nonfatal MI, PCI or CABG	> 30.6 vs. < 11.8	CVE:81; 1.53 (0.99–2.35)	Age, gender, smoking, TC, LDL-C, ApoB, hs-CRP, and and Killip class	1.0
Kardys 2012 [20]	The Netherlands	P	CAD after PCI; 161 (68)	59.4 ± 11.3	Total death, nonfatal MI, revascularization	18.2 vs. 2.7#	CVE:72; 1.53 (0.99–2.35)	age, sex, smoking, DM, hypertension, and hypercholesterolemia	6.0
Kwon 2013 [10]	Republic of Korea	R	Suspected CAD; 6252 (59.2)	61.2 ± 11.2	Cardiac death and non-fatal MI	≥20.1 vs. < 20.1	CE:100; 1.77 (1.19–2.63)	Age, gender, hypertension, DM, smoking, hyperlipidemia, and evidence of CAD at baseline coronary angiography	3.1
Li 2013 [11]	China	P	CAD; 517 (82.4)	63.4 ± 11.4	CV death, nonfatal MI, IS, revascularization	≥30 vs. < 30	CVE:102; 1.69 (1.13–2.53)	DM, previous PCI, number of lesion vessels, β-blocker use, SBP, and LVEF	2.0
Nestel 2013 [12]	Australia	R	Stable CAD; 7863 (83.1)	31–75	CV death, nonfatal MI, stroke, UA, revascularization	> 73.4 vs. ≤ 13.9	CVE:3040; 1.21(1.07–1.36)	Age, sex, treatment, stroke, DM, smoking, hypertension, TC, ApoB, apolipoprotein A1, HDL-C, timing of revascularization, SBP, AF, eGFR, BMI, dyspnea, angina grade, white blood cell count, PAD, TG, glucose, and baseline aspirin use	6.0
Guler 2013 [13]	Turkey	P	Non-ST elevation ACS;115 (70.4)	64 ± 11	CV death and ACS hospitalization	≥75 vs. < 32.2	CE:20; 2.59 (1.40–4.78)	Age, hemoglobin, creatinine, LVEF, previous MI, Killip class, and GRACE score	1.0
Park 2015 [21]	Korea	R	Angina pectoris; 595 (65.2)	63.5 ± 9.6	Total death, any MI, revascularization	≥50 vs. < 50	Total death:19; 2.47 (0.73–8.38); CV death: 7 2.85 (0.43–18.9); CVE:87; 2.09 (1.16–3.76)	Age, sex, DM, hypertension, hyperlipidemia, smoking, multivessel disease, minimal luminal diameter after PCI, reference vessel diameter after PCI, LDL-C, and total lesion length	3.0
Konishi 2015 [14]	Japan	R	Post-PCI; 411 (80.3)	67.6 ± 10.5	Total death and ACS	> 11.2 vs. < 11.2	CVE:81; 1.68 (1.03–2.70)	Age, eGFR, ACS,LVEF, multivessel disease, left anterior descending lesion, and statins	4.7
Feng 2016 [15]	China	R	Post- angiography/ PCI; 1684 (74.9)	63.3 ± 10.6	–	≥16 vs. < 16	Total death:56; 1.96 (1.07–3.59);	Age, sex, hypertension, DM, LDL-C, anemia, eGFR, LVEF, and lesion vessels	1.95
Xie 2017 [16]	China	R	Non-obstructive CAD; 451 (44.6)	58 ± 9	Cardiac death and non-fatal ACS	> 30 vs. ≤30	CE:37; 3.16 (1.60–6.23)	Age, gender, LVEF, BMI, and pre-study lipid-lowering therapies use	2.7
Suwa 2017 [17]	Japan	P	CAD; 1336 (82.0)	64.4 ± 10.2	Cardiac death and non-fatal ACS	> 21.5 vs. 21.5	CE:144; 1.28 (1.04–1.58)	Age, gender, LDL-C, HDL-C,TG, multivessel disease, ACS, and DM	4.48
Zewinger 2018 [22]	Multination	P	CAD 3313 (70)	62.7 ± 10.6	–	> 26 vs. ≤10	Total death:994; 0.95 (0.81–1.11); CV death:621 0.99 (0.81–1.2)	Age, sex, DM, SBP, BMI, smoking, eGFR, LDL-C, and use of lipid-lowering therapy.	9.9
Zhou 2018 [23]	China	P	Stable CAD; 3278 (73.4)	57.9 ± 9.8	Total death, nonfatal MI, stroke, revascularisation	≥30 vs. < 30	CVE:215; 1.02 (0.76–1.38)	Age, male, hypertension, DM, smoking, BMI, ESR, LDL-C, and hs-CRP	3.1
Shitara 2019 [18]	Japan	P	CAD with LVEF < 50%; 369 (86.7)	65.3 ± 11.7	Total death, nonfatal ACS,HF	≥21.6 vs. < 21.6	CVE:157; 1.54 (1.09–2.18)	Age, male, current smoking, chronic kidney disease, statin use, TG, DBP, LVEF, and aortic valve stenosis	5.1

*Abbreviations*: *HR* hazard ratio, *RR* risk ratio, *CI* confidence intervals, *P* prospective, *R* retrospective, *CE* cardiac events, *CVE* cardiovascular events, *BMI* body mass index, *SBP* systolic blood pressure, *LDL-C* low density lipoprotein cholesterol, *HDL-C* high-density lipoprotein cholesterol, *TG* triglycerides, *DM* diabetes mellitus, *hs-CRP* high sensitive C-reactive protein, *ApoB* apolipoprotein B, *ESR* erythrocyte sedimentation rate, *LVEF* left ventricular ejection fraction, *eGFR* estimated glomerular filtration rate, *PAD* peripheral vascular disease, *CAD* coronary artery disease, *ACS* acute coronary syndrome, *MI* myocardial infarction, *UA* unstable angina, *AF* atrial fibrillation, *IS* ischemic stroke, *HF* heart failure, *MCOS* myocardial coronary obstruction score, *LVMS* left ventricular motion scores, *TVR* target vessel revascularization, *PCI* percutaneous coronary intervention, *CABG* coronary artery bypass grafting, *CCB* calcium channel blockers. #To convert lipoprotein (a) from nmol/L to mg/mL, divide by 3.57. ^ Pooled by a fixed-effect model

### Impact of elevated lipoprotein (a) on cardiac and cardiovascular events

**Fig. 2:**
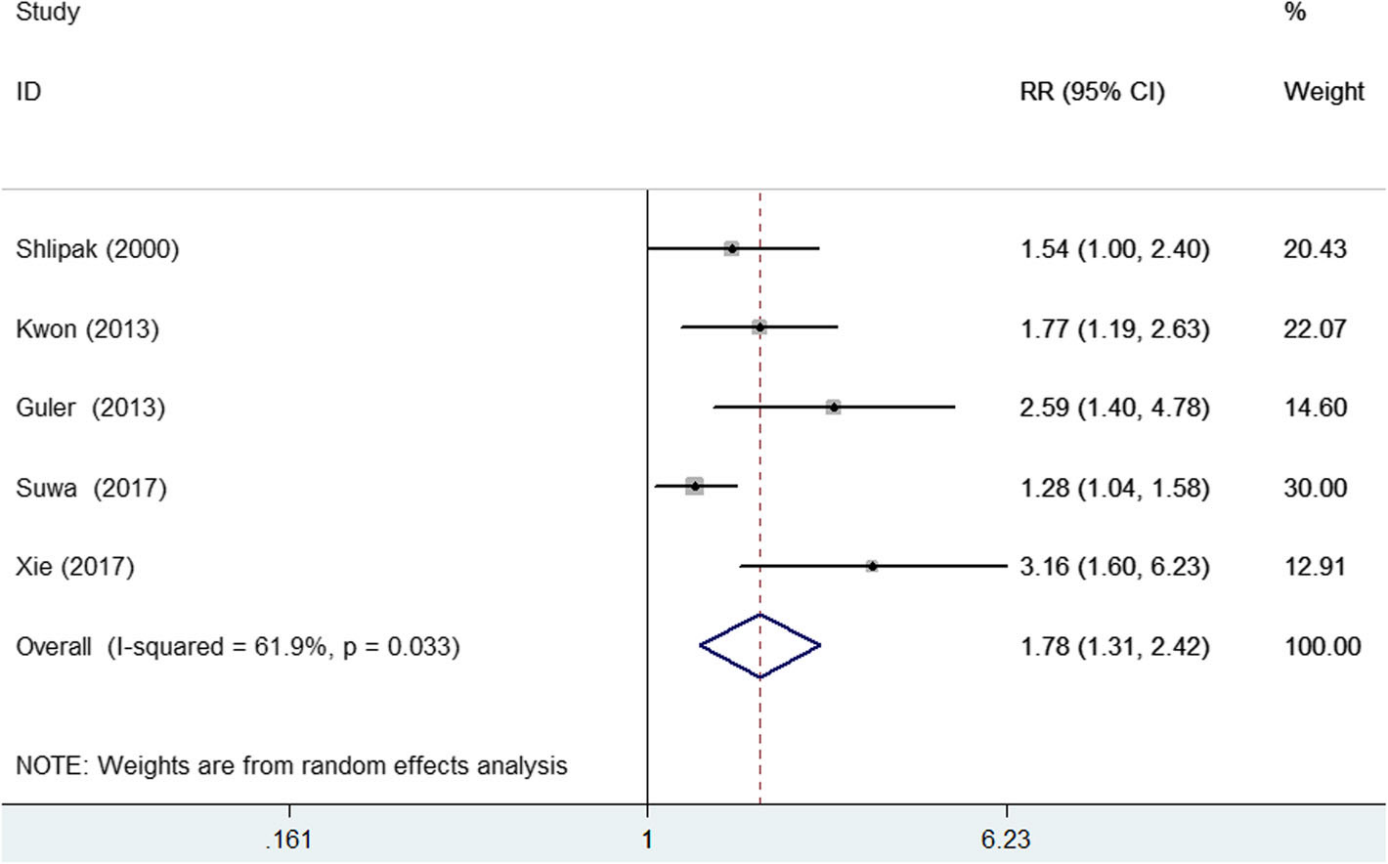
Forest plots showing pooled RR with 95% CI of cardiovascular events for the highest versus the lowest lipoprotein (a) level

Five studies [8, 10, 13, 16, 17] reported the prognostic value of elevated lipoprotein (a) level for cardiac events (Fig. 2). The pooled RR of cardiac events was 1.78 (95% CI 1.31–2.42) for the highest vs. the lowest category of lipoprotein (a) level in a random effect model, with significant heterogeneity (I^2^ = 61.9%; *p* = 0.033). Sensitivity analyses revealed no significant changes in the original pooled risk estimates when any study was excluded (data not shown).

**Fig. 3:**
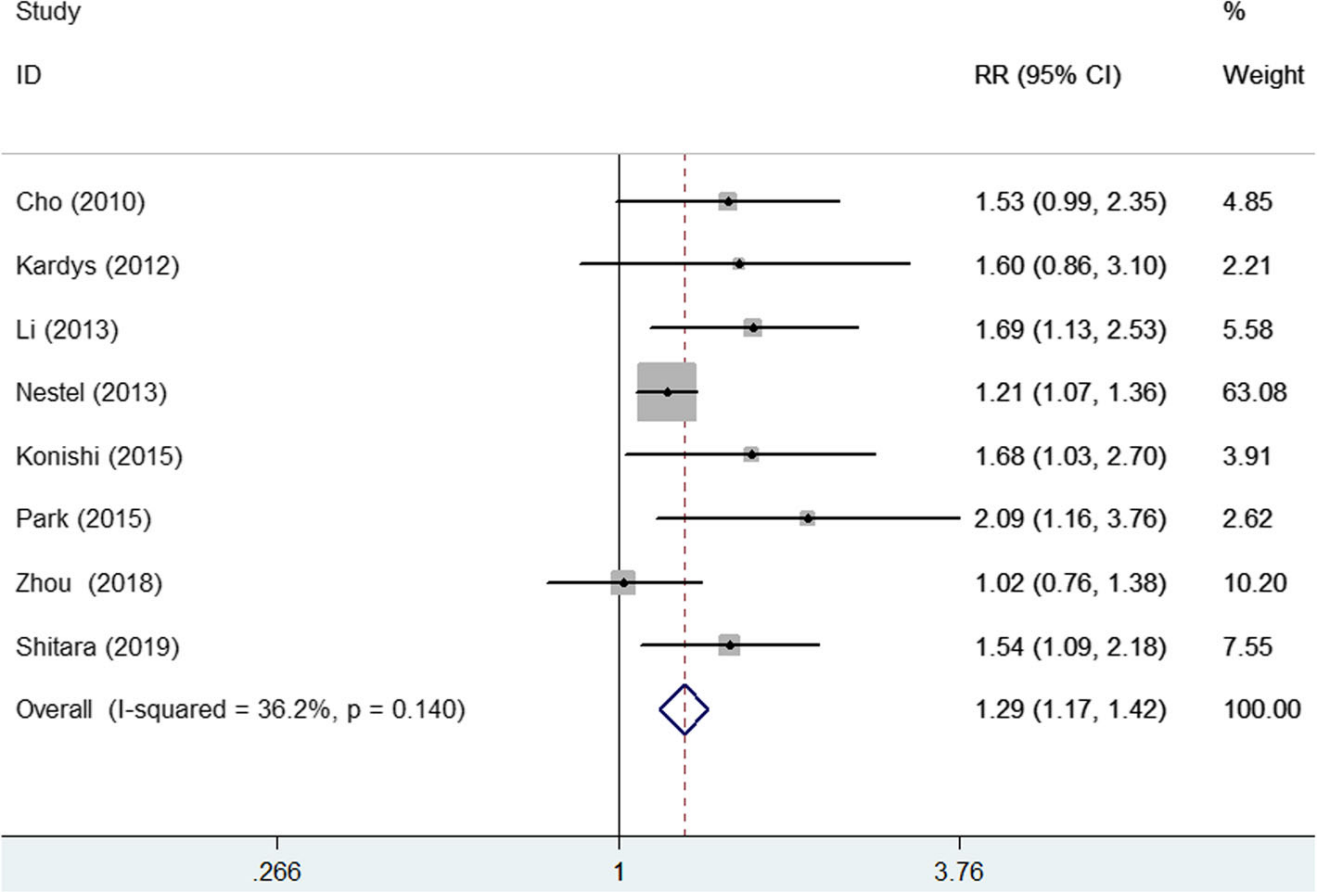
Forest plots showing pooled RR with 95% CI of cardiac events for the highest versus the lowest lipoprotein (a) level

Eight studies [11, 12, 14, 18, 19, 21, 23] reported the prognostic value of elevated lipoprotein (a) level for cardiovascular events (Fig. 3). The pooled RR of cardiovascular events was 1.29 (95% CI 1.17–1.42) for the highest vs. the lowest category of lipoprotein(a) level in a fixed-effect model, without significant heterogeneity (I^2^ = 36.2%; *p* = 0.140). Sensitivity analyses showed no significant changes in the original pooled effect sizes when any study was removed (data not shown). Subgroup analysis showed that the prognostic significance of lipoprotein (a) level for cardiovascular events was consistently found in each predefined subgroups (Additional file 2 Table S2).

### Impact of elevated lipoprotein (a) on all-cause and cardiovascular mortality

**Fig. 4:**
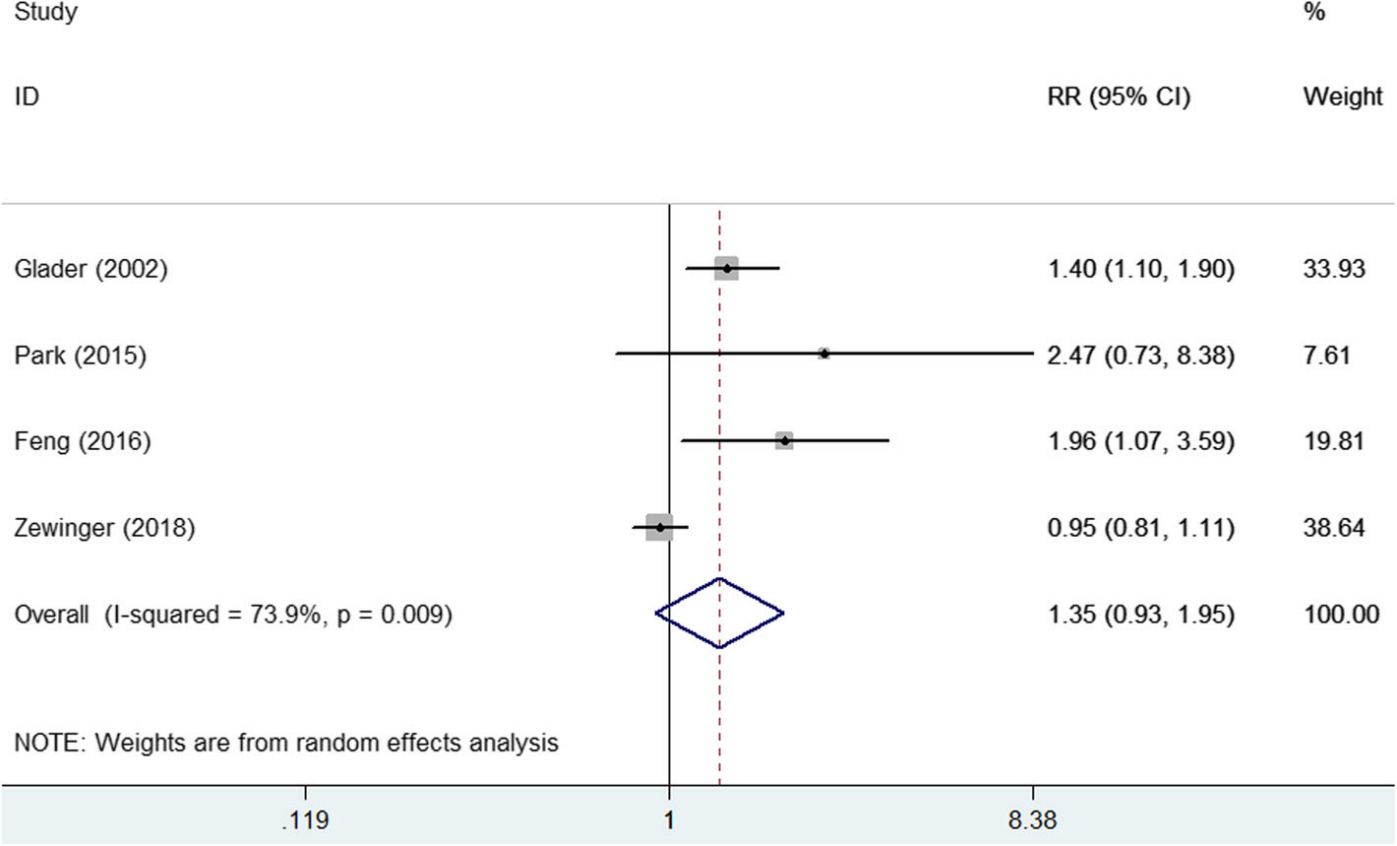
Forest plots showing pooled RR with 95% CI of all-cause mortality for the highest versus the lowest lipoprotein (a) level

**Fig. 5:**
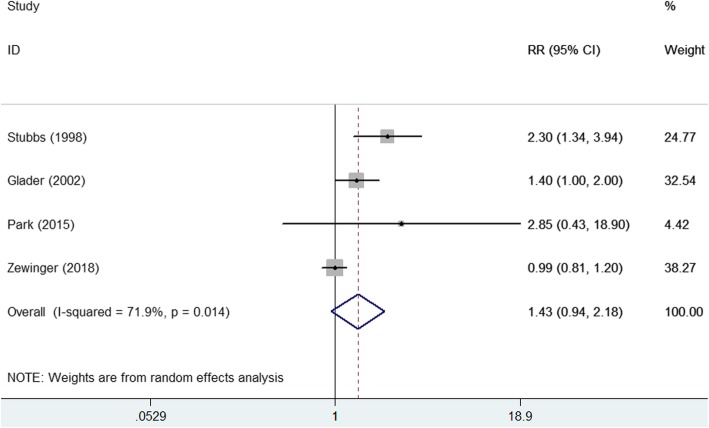
Forest plots showing pooled RR with 95% CI of cardiovascular mortality for the highest versus the lowest lipoprotein (a) level

Four studies [9, 15, 21, 22] reported the association between elevated lipoprotein(a) level and risk of all-cause mortality (Fig. 3). The pooled RR of all-cause mortality was 1.35 (95% CI 0.93–1.95) for the highest vs. the lowest category of lipoprotein (a) level in a random effect model, with significant heterogeneity (I^2^ = 73.9%; *p* = 0.009). Four studies [7, 9, 21, 22] reported the association between elevated lipoprotein(a) level and risk of cardiovascular mortality (Fig. 4). The pooled RR of cardiovascular mortality was 1.43 (95% CI 0.94–2.18) for the highest vs. the lowest category of lipoprotein (a) level in a random effect model (Fig. 5), without significant heterogeneity (I^2^ = 71.9%; *p* = 0.014). Sensitivity analyses by removing any study at each time slightly changed the original pooled effect sizes of all-cause and cardiovascular mortality (data not shown).

### Publication bias

The funnel plot is potentially unreliable for less than the recommended arbitrary minimum number of 10 studies analyzed [26], so we did not construct the funnel plot to examine publication bias.

## Discussion

The main findings of this meta-analysis is that an elevated lipoprotein (a) level is an independent predictor of cardiac and cardiovascular events in CAD patients. These patients with the highest lipoprotein (a) level exhibited approximately 90 and 28% higher risk of cardiac and cardiovascular events, respectively, than those with the lowest lipoprotein (a) level. However, elevated baseline lipoprotein (a) level was not associated with an increased risk of cardiovascular and all-cause mortality in CAD patients.

The results presented our study are quite consistent with those of previous meta-analysis [27] showed that elevated lipoprotein (a) level was significantly associated with an increased risk of cardiovascular events in patients with established CAD. However, this well-designed meta-analysis did not evaluate the association of lipoprotein (a) with cardiovascular and all-cause mortality. Another individual patient-data meta-analysis [28] indicated that elevated baseline lipoprotein (a) exhibited an independent approximately linear association with cardiovascular events in patients with established cardiovascular disease. Consistent with previous meta-analyses, our study confirmed the prognostic role of lipoprotein (a) for predicting cardiovascular events in CAD patients. Additionally, analysis of lipoprotein (a) level by continuous variables further supported the prognostic significance of elevated lipoprotein (a) level for predicting cardiac and cardiovascular events in these patients [17, 29]. Elevated lipoprotein (a) level has been demonstrated to correlate with a higher mortality rate not only in subjects at high and intermediate risk, but also in young women with a low cardiovascular risk profile [30].

Our subgroup analysis further supported the prognostic value of lipoprotein (a) level for predicting cardiovascular events. There was a close association between elevated lipoprotein(a) level and higher risk cardiovascular events in the subgroup with follow-up duration < 2 years in the current meta-analysis, suggesting that the prognostic value of lipoprotein (a) level weakened with the lengthening of the follow-up duration. Kardys’ study [20] also showed that lipoprotein (a) was associated with a 3.1-fold increased 1-year risk of cardiovascular events but lost significance with long-term follow-up. CAD is a heterogeneous condition, which spans from stable clinical condition to acute myocardial infarction. Notably, the effect of elevated lipoprotein (a) level on clinical outcomes was different between patients with and without ACS [18]. However, we could not perform subgroup analysis by subtype of CAD due to insufficient data.

Despite attaining the target low-lipid profile, a number of CAD patients remain at risk for cardiovascular events [31]. Elevated lipoprotein (a) level may account for some of the residual cardiovascular risk. Lipoprotein (a) could predict worse clinical outcomes even for patients with CAD who achieved target lipid level [14]. Importantly, determination of lipoprotein (a) level may also help to identify patients who require more intensive treatment. Future studies are warranted to evaluate whether lowering lipoprotein (a) level can offer cardiovascular benefits in patients with CAD.

Both life-style improvement and available lipid-lowering drugs seem to poorly affect blood levels of lipoprotein (a) [32, 33]. Mild reduction achievable with PCKS9 inhibitors or niacin seems not to be related to reduce cardiovascular events [34]. Therefore, there are still unmet clinical demands in the management of dyslipidemia.

The mechanisms accounting for predictive value of lipoprotein (a) among CAD patients remain unclear. One potential explanation may be lipoprotein (a) mediating proinflammatory and antifibrinolytic effects [35]. Another explanation is that elevated lipoprotein (a) can damage endothelial and anticoagulant function by promoting endothelial dysfunction and increasing phospholipid oxidation [36, 37].

Several potential limitations of this meta-analysis should be acknowledged. First, this is not an individual-level meta-analysis and patients’ characteristics may have potential to affect the pooling results. Second, the cutoff value of elevated blood lipoprotein (a) level varied across studies and we could not define the appropriate cutoff value of lipoprotein (a) elevation. Third, moderate heterogeneity was observed in the pooling cardiac events and all-cause mortality. The heterogeneity may be partly explained by the subtype of CAD, study design, follow-up duration, methods for measuring lipoprotein (a), and cutoff value of lipoprotein(a) elevation. Fourth, due to insufficient data from the eligible studies, we failed to evaluate the prognostic role of lipoprotein (a) level by continuous data due to insufficient studies. Finally, we did not construct the funnel plot for detecting publication bias due to the analyzed studies was less than the recommended arbitrary minimum number of 10.

## Conclusions

The current meta-analysis indicates that elevated lipoprotein (a) level is an independent predictor of cardiac and cardiovascular events in CAD patients. As for lipoprotein(a) level is less affected by lifestyle, diet and medical therapy, measurement of lipoprotein (a) level has potential to improve the risk stratification CAD patients. Future well-designed studies are warranted to investigate whether the prognostic utility of lipoprotein(a) level is different subtype CAD patients.

## Additional files

Additional file 1: **Table S1.** Quality assessment of the included studies (DOC 64 kb)

Additional file 2: **Table S2.** Subgroup analyses on cardiovascular events (DOC 41 kb)

## Data Availability

All data generated or analyzed during this study are included in this article.

## Abbreviations

CAD: Coronary artery disease
CI: Confidence interval
HR: Hazard ratio
NOS: Newcastle-Ottawa Scale
OR: Odds ratio
RR: Risk ratio
