# Supplementary material for: Prognostic value of lipoprotein (a) level in patients with coronary artery disease: a meta-analysis
Source: Lipids Health Dis. 2019 Jul 8;18:150. doi: 10.1186/s12944-019-1092-6 (PMC6615167; doi:10.1186/s12944-019-1092-6)
Supplement: Supplementary file 1 — Table S1. Quality assessment of the included studies (DOC 64 kb) [file 12944_2019_1092_MOESM1_ESM.doc]

**~~Table S1 Quality assessment of the included studies~~**

| Author/Year | Representativeness of the exposed cohort | Selection of the non exposed cohort | Ascertainment of exposure | Demonstration that outcome was not present at study start | Comparability of cohorts on the basis of the design or analysis | Assessment of outcome | Enough follow-up periods(> 2 years) | Adequacy of follow-up of cohorts | Total NOS |
| --- | --- | --- | --- | --- | --- | --- | --- | --- | --- |
| Stubbs 1998 [7] | ★ | ★ | ★ | ★ | ★ | ★ | ★ |  | 7 |
| Shlipak 2000 [8] |  | ★ | ★ | ★ | ★ | ★ | ★ | ★ | 8 |
| Glader 2002 [9] | ★ | ★ | ★ | ★ | ★ | ★ | ★ | ★ | 8 |
| Cho 2010 [19] |  | ★ | ★ | ★ | ★ | ★ |  | ★ | 6 |
| Kardys 2012 [20] | ★ | ★ | ★ | ★ | ★ | ★ | ★ | ★ | 8 |
| Kwon 2013 [10] |  | ★ | ★ | ★ | ★ | ★ | ★ | ★ | 7 |
| Li 2013 [11] | ★ | ★ | ★ | ★ | ★ | ★ |  | ★ | 7 |
| Nestel 2013 [12] | ★ | ★ | ★ | ★ | ★ | ★ | ★ | ★ | 8 |
| Guler 2013 [13] |  | ★ | ★ | ★ | ★★ | ★ |  | ★ | 7 |
| Park 2015 [21] | ★ | ★ | ★ | ★ | ★ | ★ | ★ | ★ | 8 |
| Konishi 2015 [14] |  | ★ | ★ | ★ | ★ | ★ | ★ | ★ | 7 |
| Feng 2016 [15] |  | ★ | ★ | ★ | ★★ | ★ |  |  | 6 |
| Xie 2017 [16] |  | ★ | ★ | ★ | ★ | ★ | ★ | ★ | 7 |
| Suwa 2017 [17] | ★ | ★ | ★ | ★ | ★★ | ★ | ★ | ★ | 9 |
| Zewinger 2018 [22] | ★ | ★ | ★ | ★ | ★ | ★ | ★ | ★ | 8 |
| Zhou 2018 [23] | ★ | ★ | ★ | ★ | ★ | ★ | ★ | ★ | 8 |
| Shitara 2019 [18] |  | ★ | ★ | ★ | ★ | ★ | ★ | ★ | 7 |

NOS, Newcastle-Ottawa Scale.
