# Supplementary material for: Prognostic value of lipoprotein (a) level in patients with coronary artery disease: a meta-analysis
Source: Lipids Health Dis. 2019 Jul 8;18:150. doi: 10.1186/s12944-019-1092-6 (PMC6615167; doi:10.1186/s12944-019-1092-6)
Supplement: Supplementary file 2 — Table S2. Subgroup analyses on cardiovascular events (DOC 41 kb) [file 12944_2019_1092_MOESM2_ESM.doc]

~~Table S2 Subgroup analyses on cardiovascular events~~

| Subgroup | Number of studies | Pooled risk ratios | 95% confidence intervals | Heterogeneity between studies |
| --- | --- | --- | --- | --- |
| Study design  Prospective  Retrospective | 3  5 | 1.26  1.37 | 1.12–1.41  1.15–1.62 | p=0.098; I^2^ =57.0%  p=0.222; I^2^ =30.0% |
| Sample sizes  <1000  >1000 | 6  2 | 1.64  1.18 | 1.37–1.97  1.06–1.32 | p=0.969; I^2^ =0.0%  p=0.298; I^2^ =7.8% |
| Ethnicity  Asian  Caucasian | 6  2 | 1.43  1.22 | 1.22–1.68  1.09–1.37 | p=0.162; I^2^ =36.6%  p=0.401; I^2^ =0.0% |
| Follow-up duration  >2 years  ≤2 years | 6  2 | 1.26  1.61 | 1.14–1.39  1.20–2.17 | p=0.136; I^2^ =40.5%  p=0.741; I^2^ =0.0% |
| Adjusting statins use  Yes  No | 2  6 | 1.59  1.26 | 1.20–2.10  1.14–1.39 | p=0.774; I^2^ =0.0%  p=0.128; I^2^ =41.6% |
| Adjusting lipids  Yes  No | 6  2 | 1.25  1.69 | 1.13–1.39  1.24–2.30 | p=0.168; I^2^ =35.9%  p=0.985; I^2^ =0.0% |
